# Analysis of Pollination Process between Flowers and Honeybees to Derive Insights for the Design of Microrobots

**DOI:** 10.3390/biomimetics9040235

**Published:** 2024-04-15

**Authors:** Pratap Sriram Sundar, Chandan Chowdhury, Sagar Kamarthi

**Affiliations:** 1 Munjal Institute for Global Manufacturing and Punj Lloyd Institute of Infrastructure Management, Indian School of Business, Mohali 140306, India; pratap_sundar@isb.edu; 2 Munjal Institute for Global Manufacturing and Punj Lloyd Institute of Infrastructure Management, Indian School of Business, Gachibowli, Hyderabad 500111, India; chandan_chowdhury@isb.edu; 3 College of Engineering, Northeastern University, 360 Huntington Avenue, Boston, MA 02115, USA

**Keywords:** pollination process, honeybees, flowers, axiomatic design, nature-inspired design, design evaluation, sustainability, microrobots

## Abstract

Pollination is a crucial ecological process with far-reaching impacts on natural and agricultural systems. Approximately 85% of flowering plants depend on animal pollinators for successful reproduction. Over 75% of global food crops rely on pollinators, making them indispensable for sustaining human populations. Wind, water, insects, birds, bats, mammals, amphibians, and mollusks accomplish the pollination process. The design features of flowers and pollinators in angiosperms make the pollination process functionally effective and efficient. In this paper, we analyze the design aspects of the honeybee-enabled flower pollination process using the axiomatic design methodology. We tabulate functional requirements (FRs) of flower and honeybee components and map them onto nature-chosen design parameters (DPs). We apply the “independence axiom” of the axiomatic design methodology to identify couplings and to evaluate if the features of a flower and a honeybee form a good design (i.e., uncoupled design) or an underperforming design (i.e., coupled design). We also apply the axiomatic design methodology’s “information axiom” to assess the pollination process’s robustness and reliability. Through this exploration, we observed that the pollination process is not only a good design but also a robust design. This approach to assessing whether nature’s processes are good or bad designs can be valuable for biomimicry studies. This approach can also inform design considerations for bio-inspired innovations such as microrobots.

## 1. Introduction

This research aims to learn from the design features of flowers and pollinators in angiosperms that make the pollination process functionally effective and efficient. Our approach in this paper is to analyze the design aspects of pollination by flowers and honeybees using the axiomatic design methodology. In axiomatic design, functional requirements (FRs) are the essential outcomes that a system must achieve, while design parameters (DPs) are the physical or procedural means through which these outcomes are realized. The principle that FRs need to be satisfied independently by DPs is grounded in the first axiom of axiomatic design, known as the independence axiom. The second axiom, known as the information axiom, helps select if multiple designs satisfy the first axiom. The simple design with minimum information content is robust and reliable among the alternative designs that meet the independent axiom. The requirement for FRs to be satisfied independently by DPs underpins the goal of creating designs that are simple, efficient, and capable of meeting user needs without unnecessary complexity or interdependence. By adhering to the independence and information axioms, designers can create systems that are effective, adaptable, and robust against changes and uncertainties.

For the pollination process, DPs for FRs of flowers and honeybees have already been determined by nature through coevolution over millions of years. Scientists have discovered FRs of flowers and honeybees through their observations and research over centuries. This analysis presented in this paper maps the FRs onto DPs to check for the couplings.

Pollination is a vital ecosystem service, required by 76% of global crops and an estimated 87.5% of all flowering plants. The western honeybees (*Apis mellifera* L.) are the primary crop pollinator species globally, providing roughly 50% of global crop pollination. Honeybees pollinate about one hundred plant families, such as strawberries, apples, pomegranates, beans, sunflowers, cotton, coffee plants, sesame, and okra [1]. Honeybees have long been associated with humans and are the most widely domesticated pollinator species globally [2]. Pollination plays a pivotal role in maintaining biodiversity and the health of natural ecosystems. It promotes genetic diversity within plant populations, enabling adaptation and resilience in the face of environmental changes. Pollinators like wind, water, insects, birds, bats, mammals, amphibians, and mollusks facilitate plant reproduction. In the lingua of botany, these pollination processes are called anemophily (wind pollination), hydrophily (water pollination), zoophily (animal pollination), ornithophily (bird pollination), chiropterophily (bat pollination), malacophily (slug and snail pollination), and entomophily (insect pollination). Proctor [3] presented a detailed account of the pollination process. The pollination process is a vital link in the food chain of fruits, vegetables, and seeds, which feed animals, birds, and humans. In agricultural systems, pollination is indispensable for crop production. Pollinators enhance crop yields, improve fruit quality, and increase seed production. Many economically important crops, such as fruits, vegetables, nuts, and oilseeds, rely heavily on pollinators. The economic value of pollination services is estimated to be billions of dollars annually, highlighting the direct impact of pollinators on global food security and livelihoods. The pollination process showcases the intricate interdependencies and harmonious coexistence in nature. The symbiotic relationship between plants and pollinators highlights the profound significance of biodiversity conservation and sustainable environmental practices.

Through the axiomatic design methodology, this study observed that the pollination process involving flowers and honeybees is a good and robust design. Inspired by the design of flowers and honeybees, this study indicated design features for microrobots, particularly for cradle-to-cradle life cycle, material choices, power source, miniaturization, sensors, actuators, manufacturing techniques, and upcycle.

## 2. Analysis

In this work, we analyze the anatomy and functionality of flowers and honeybees using the first axiom, i.e., the independence axiom of the axiomatic design methodology, which is applied to test for couplings [4]. This axiom checks if FRs are independently satisfied by DPs, i.e., a dedicated DP serves each FR. FRs are the essential outcomes that a system must achieve, while DPs are the physical or procedural means through which these outcomes are realized. When multiple FRs share a common DP, the latter creates a coupling among the former, and changes made to the DP significantly impact two or more FRs. The designs that pass the axiomatic design filter will offer enhanced performance. Optimizing each design aspect becomes more straightforward when each FR is independently satisfied by a specific DP. Designers can adjust or improve individual DPs to better meet their corresponding FRs without unintended consequences for other system parts. This targeted optimization can lead to overall better system performance.

Axiomatic design introduces design matrix-based analysis to assess and mitigate couplings in FRs. There are three types of designs: (1) uncoupled design, (2) decoupled design, and (3) coupled design. In an uncoupled design, we determine FR-specific DPs such that the DPs satisfy their corresponding FRs without cross interference. In a decoupled design, we can determine DPs such that they satisfy FRs independently but with a constraint that we must realize DPs in a specific order. When we cannot determine DPs that satisfy FRs independently, we end up with a coupled design in which a DP satisfies multiple FRs. In a coupled design, changes made to a DP to improve its effectiveness to satisfy an FR may have unintended consequences on other connected FRs. The second axiom of the methodology, known as the information axiom, emphasizes the selection of an informationally lean and functionally reliable alternative among the design solutions that satisfy the independent axiom. According to the axiomatic design methodology, the best design is a functionally uncoupled design with minimum information content; conversely, a coupled design with redundant information is the least desirable outcome [5].

$$\left\{\begin{array}{c} {\mathrm{FR}}_{1}\\ {\mathrm{FR}}_{2}\\ {\mathrm{FR}}_{3}\\ \vdots \\ {\mathrm{FR}}_{n} \end{array}\right\}\overset{\mathrm{Satisfied}\;\mathrm{by}}{\leftarrow }\left[\begin{array}{ccccc} a_{11} & a_{12} & a_{13} & \cdots & a_{1n}\\ a_{21} & a_{22} & a_{23} & \cdots & a_{2n}\\ a_{31} & a_{32} & a_{33} & \cdots & a_{3n}\\ \vdots & \vdots & \vdots & \ddots & \vdots \\ a_{n1} & a_{n2} & a_{n3} & \cdots & a_{nn} \end{array}\right]\left\{\begin{array}{c} {\mathrm{DP}}_{1}\\ {\mathrm{DP}}_{2}\\ {\mathrm{DP}}_{3}\\ \vdots \\ {\mathrm{DP}}_{n} \end{array}\right\}\;\mathrm{where}\;a_{ij}=\{\begin{array}{c} 1{\;\mathrm{if}\;\mathrm{DP}}_{j}{\;\mathrm{satisfies}\;\mathrm{FR}}_{i}\\ 0{\;\mathrm{if}\;\mathrm{DP}}_{j}{\;\mathrm{does}\;\mathrm{not}\;\mathrm{satisfy}\;\mathrm{FR}}_{i}\; \end{array}$$

$$\left\{{\mathrm{FR}}_{i}\right\}\overset{\mathrm{Satisfied}\;\mathrm{by}}{\leftarrow }\left\{\overset{n}{\underset{j=1}{\cup }}a_{ij}{\mathrm{DP}}_{j}\right\}=\left\{\begin{array}{c} a_{i1}{\mathrm{DP}}_{1}\\ \vdots \\ a_{ij}{\mathrm{DP}}_{j}\\ \vdots \\ a_{in}{\mathrm{DP}}_{n} \end{array}\right\}$$

The design matrix is square with binary elements; *a_ij_* = 1 indicates that DP*_j_* satisfies or influences FR*_i_*, and *a_ij_* = 0 indicates that DP*_j_* has no association with FR*_i_*. The design matrix of an uncoupled design will have 1s only in the diagonal positions and 0s in the other positions. The design matrix of a decoupled design will have 1s in the diagonal positions and the upper or lower triangle positions. The design matrix of coupled design will have 1s in the diagonal positions and both in the upper and lower triangle positions.

The uncoupled design and simple design ensure functional efficiency and robustness. This one-to-one correspondence simplifies the design by making it clear which DP is responsible for which FR. It makes the design more robust, as changes or failures in one part of the system do not directly compromise the performance of others. A design adhering to the two axioms is easier to maintain and troubleshoot because the relationship between FRs and DPs is clear and straightforward. If an FR is not being met, the corresponding DP can be directly identified and assessed for issues, making the maintenance process more efficient. An uncoupled design supports system scalability. As the system grows or its requirements change, new FRs can be addressed by introducing new DPs without redesigning existing system components. This modularity is a key advantage in evolving systems. A coupled design makes systems fragile, where changes to one element cause cascading effects on others. By ensuring FRs are met independently by DPs, axiomatic design minimizes coupling, leading to more flexible and resilient designs.

As mentioned earlier in this paper, DPs for FRs of flowers and honeybees have already been determined by nature through coevolution over millions of years. Still, scientists have been discovering FRs and DPs for the past few centuries.

Section 3 of this paper presents the information axiom [6], which gives a criterion for selecting a robust design among all candidate designs screed by the independent design.

## 3. Pollination Process

A typical manufacturing company supply chain comprises material, information, and cash flow. A supply chain has many relationships between customers and suppliers in a company’s ecosystem. Often, many nodes in a supply chain play a dual role of being both the customer and the supplier. We observe a similar supply chain in an ecosystem of flowering plants and honeybees. Honeybees collect water, nectar, pollen, propolis (resins), oil, and fragrances (scents) from plants. This collection and transportation are like material flow in a supply chain. In a honeybee ecosystem, a complex communication system used by honeybees facilitates information flow. Honeybees need nectar and pollen to feed, survive, and grow the colony. Honeybees make bee bread of pollen with a mix of nectar or honey and a little saliva [7]. The honeybee is a customer in a natural ecosystem, and the flower is the supplier. In a reverse scenario, a plant needs pollination to produce seeds and fruits, and honeybees provide service by fulfilling this function as they collect honey from flowers. In this transaction, a plant/flower is the customer, and a honeybee is the service provider. The partnership between flowers and honeybees is a balanced pollination mutualism and a win–win situation [8]. The symbiotic interaction between plants/flowers and pollinators (i.e., honeybees) can take six different paths depending on “reward collection without pollination service” and “pollination service without reward collection”: (1) primary or secondary honey and pollen collection without pollination; (2) reward harvesting with low pollination efficiency; (3) balanced pollination mutualism; (4) pollination with little reward; (5) pollination by trapping pollinators for an extended time; and (6) pollination through deception without reward. Hung et al. [9] used a global dataset of 80 published plant–pollinator interaction networks and pollinator effectiveness measures from 34 plant species to assess the importance of *Apis mellifera* in natural habitats. *Apis mellifera* is the most frequent floral visitor in natural habitats worldwide, averaging 13% of floral visits across most of the plant–pollinator interaction networks, with 5% of plant species recorded as being exclusively visited by *Apis mellifera*. Research by Chambo et al. [10] showed that honeybees significantly pollinate sunflower plants, yielding 43% higher seed production.

The pollination process ensures the transfer of pollen grains, allowing the fertilization and subsequent formation of fruits, seeds, and new generations of plants. Willmer [11] explained how bees enable the pollination process using their basitarsal brush, a collection of hair on the last segment of the foreleg. As a bee forages, its face rubs the anthers of the flower, accumulating pollen all over its head. The bee then uses the brush on its legs to remove the pollen on its head and place it on structures designed for pollen transport [12].

Honeybees rely on several interacting navigational mechanisms [13], including (1) cues from odors, (2) a measure of distance over the ground, (3) direction relative to the pattern of UV light in the sky, (4) the Sun’s position, and (5) recognition of places or landmarks. Honeybees must fly to food sources, find and transport food (pollen and nectar), and communicate with other bees. Honeybees use the Sun’s position, the Earth’s magnetic field, an internal biological clock, and an odometer to accomplish these activities. Dyer and Gould [14] described a scientific account of a honeybee’s ability to find its way that depends on a hierarchy of sophisticated orientation mechanisms, such as the Sun’s visible light, ultraviolet rays, and landmarks. Ribi et al. [15] described the construction and arrangement of rhabdoms in the ocelli of a honeybee, suggesting that ocelli play a role in altitude control and sensing skylight polarization compass information. Collett [16] explained how honeybees remember visual panoramas in a compass-based coordinate frame, linking stored visual features of the panorama and signals from their sun-based compass. Dacke and Srinivasan [17] suggested that honeybees may possess two different odometers: a “community odometer” that provides information to nestmates by performing the dance and a “personal odometer” that experienced bees use to return to a previously visited source. Honeybees might also use a celestial compass. Najera et al. [18] discovered that the chosen departure direction at a secondary hub could be guided exclusively by either celestial or terrestrial compass cues. Honeybees navigate in three-dimensional space like many living things. Dacke and Srinivasan [19] found that the odometric signal depends only upon the total distance traveled along the path and is independent of its three-dimensional configuration.

The honeybee nervous system facilitates information processing and decision making. The brain of a honeybee, which is about the size of a sesame seed, plays a central role. Nobel-Prize-winning insect neuroanatomist Santiago Ramón y Cajal conducted pioneering studies of insect nervous systems. He created a schematic diagram of the complex anatomy of neurons in a honeybee’s retina and optic lobe [20]. Chittka [21] described the anatomy of a honeybee brain and its functions, such as learning, visual information processing, and navigation. Rueppell et al. [22] estimated the life expectancy of honeybees (*Apis mellifera* L.) based on three large-scale experiments. Worker honeybees spend their initial days of adult life working in the nest, then transition to foraging and die between 4 and 8 weeks of age. Scientists believe foraging is the primary reason for the early death of worker bees.

Kirchner and Towne [23] explained how honeybees use their dance language to communicate. Karl von Frisch [24] won the Nobel Prize in Medicine in 1973 for his contributions to ethology, particularly the “dance language” of honeybees to direct other bees to nectar-rich areas. Frisch [25] described the waggle dance and other forms of honeybee communication. Frisch [26] presented a comprehensive picture of the honeybee colony, including how honeybees build a hive, care for the brood, swarm in the colony, and organize division of labor. In his book, he also covered various aspects of honeybees’ life, including their smell, taste, eyes, vision, orientation during navigation, sense of time, mental capacity, and contribution to agriculture. Fernando et al. [27] developed a recording setup and software for automatically recognizing individually tagged bees and decoding dances to improve measurement precision, remove human bias, and accelerate data collection. In addition to their dance, honeybees use their pheromones for advanced communication. Bortolotti and Costa [28] described queen and worker bee pheromones that play a vital role in communication inside and outside the hive.

Forager bees fly as far as 14 km (8.7 miles) to a patch of flowers, gather a load of nectar, pollen, or both, and then fly home, where they quickly offload their food and then head out on the next collecting trip. Seely [29] analyzed the distribution of distances to a colony’s forage sites based on 1871 waggle dances. Honeybees use vision, colors, scents, floral guides, and electrostatic forces to locate a food source. Pollinators use floral scent during foraging to identify and discriminate flowers. Honeybees can discriminate the scents of different flowers. The experiments by Wright et al. [30] showed that honeybees use floral volatiles to distinguish subtle differences in scent. Dinkel et al. [31] investigated floral guides that help bees locate food sources. Clarke et al. [32] studied the electrostatic forces between a flower and a bee that aid pollen transfer. Vaknin et al. [33] developed two mathematical models that describe the complex system of the charged body of a honeybee approaching a flower.

The pollination process has three steps: navigation, localization, and communication. In the following, we describe design matrices that relate FRs and DPs to examine the applicability of the independence axiom in the pollination process. Often, the DPs are dual. For example, honeybees use the Sun’s visible light and invisible ultraviolet spectrum navigation. Similarly, the Earth’s magnetic field and the internal compass of a honeybee work together to help bees navigate. The scent of a flower and the olfactory system of a honeybee work together in homing into a suitable flower. Honeybees use their legs to land on flower petals successfully. The positive electrostatic charge on the body of a honeybee and the negative electrostatic charge on a flower facilitate pollen transfer.

### 3.1. Coevolution of Flowers and Honeybees

Coevolution is the reciprocal genetic change in interacting species, owing to natural selection imposed on each other. The nature and strength of an interaction between two species may vary depending on genotype, environmental conditions, and other species with which those species interact [34]. The mutualistic relationship between flowers and honeybees is a remarkable example of coevolution, where two species influence each other’s evolution over time. Flowers have evolved complex structures, colors, and scents to attract pollinators [35]. The honeybees have developed unique adaptations to gather nectar and pollen efficiently. The coevolutionary process between flowers and honeybees has led to changes in floral characteristics to enhance pollination efficiency. Scientific studies have shown that specific traits, such as the shape of the corolla, length of floral tubes, and arrangement of reproductive structures, have coevolved to match the mouthparts, body size, and behavior of honeybees [36].

The pollination process of flowers by honeybees is not unique. Many other flower–pollinator combinations besides honeybees play crucial roles in pollination, and some may even be more effective in specific contexts, such as bumblebees that buzz pollinate certain flowers, moths that pollinate flowers that bloom at night, and beetles for certain plants that are attracted to flowers with a strong scent. The effectiveness of a pollinator depends on various factors, including the flower’s characteristics, the pollinator’s behavior, and the ecological context. In diverse ecosystems, a combination of different pollinators ensures the pollination of various plant species. However, this paper focuses only on the pollination process of flowers that honeybees visit.

**Figure 1:**
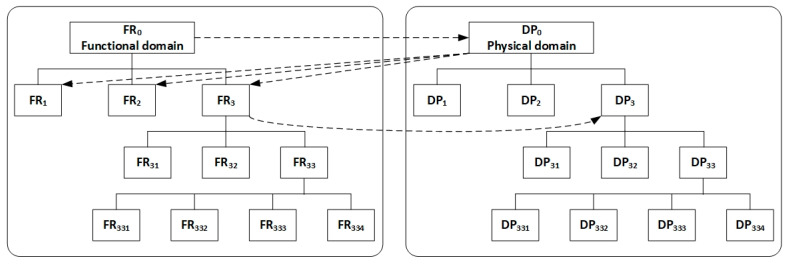
Zigzagging to decompose FRs and DPs of the pollination process to represent a hierarchical coevolutionary process.

Design hierarchy is created through a process called zigzagging in axiomatic design technology. FRs and DPs must be decomposed into a hierarchy until a detailed design is complete [37]. The details of FRs and DPs of the communication system between a honeybee and a flower are given in Table 1. Coevolution must have zigzagged working between the domains of FRs and DPs of both a flower and a honeybee. This section does not present a complete zigzag diagram due to space limitations, but a partial picture of the zigzagging of the communication system is shown in Figure 1.

### 3.2. Independence Axiom of Navigation System

The following are the critical FRs of a honeybee and a flower that facilitate pollination:

Navigation
FR_1_ = Enable navigation.
  FR_11_ = Guide by visible light.
  FR_12_ = Guide by invisible light.
  FR_13_ = Measure magnetic north.
  FR_14_ = Measure distance.
  FR_15_ = Measure time.
Localization (Homing in)
FR_2_ = Home in on specific feature of a flower.
  FR_21_ = Guide by scent.
  FR_22_ = Locate flower.
  FR_23_ = Land on flower.
  FR_24_ = Attract pollen.
  FR_25_ = Locate nectary.
Communication
FR_3_ = Enable communication.
  FR_31_ = Mechanical communication.
  FR_32_ = Chemical communication.
  FR_33_ = Dance communication.
  FR_331_ = Location of food source near the hive.
  FR_332_ = Location of food source far from the hive.
  FR_333_ = Distance to the food source.
  FR_334_ = Direction of navigation.

Given below are DPs that satisfy the FRs of flowers and honeybees. Table 2 shows the master design matrix of the overall pollination. In this section, we map the FRs onto DPs to check for the couplings.

$$\begin{array}{c} \left\{\begin{array}{c} {\mathrm{FR}}_{1}\\ {\mathrm{FR}}_{2}\\ {\mathrm{FR}}_{3} \end{array}\right\}\overset{\mathrm{Satisfied}\;\mathrm{by}}{\leftarrow }\left[\begin{array}{ccc} 1 & 0 & 0\\ 0 & 1 & 0\\ 0 & 0 & 1 \end{array}\right]\left\{\begin{array}{c} \mathrm{Navigation}\;\mathrm{system}\\ \mathrm{Localization}\;\mathrm{system}\\ \mathrm{Communication}\;\mathrm{system} \end{array}\right\}\\ \left\{\begin{array}{c} {\mathrm{FR}}_{11}\\ {\mathrm{FR}}_{12}\\ {\mathrm{FR}}_{13}\\ {\mathrm{FR}}_{14}\\ {\mathrm{FR}}_{15} \end{array}\right\}\overset{\mathrm{Satisfied}\;\mathrm{by}}{\leftarrow }\left[\begin{array}{ccccc} 1 & 0 & 0 & 0 & 0\\ 0 & 1 & 0 & 0 & 0\\ 0 & 0 & 1 & 0 & 0\\ 0 & 0 & 0 & 1 & 0\\ 0 & 0 & 0 & 0 & 1 \end{array}\right]\left\{\begin{array}{c} \mathrm{Sun}’s\;\mathrm{visible}\;\mathrm{light}\\ \mathrm{Sun}’s\;\mathrm{ultraviolet}\;\mathrm{light}\\ \mathrm{Internal}\;\mathrm{compass}\\ \mathrm{Odometer}\\ \mathrm{Internal}\;\mathrm{clock} \end{array}\right\}\\ \left\{\begin{array}{c} {\mathrm{FR}}_{21}\\ {\mathrm{FR}}_{22}\\ {\mathrm{FR}}_{23}\\ {\mathrm{FR}}_{24}\\ {\mathrm{FR}}_{25} \end{array}\right\}\overset{\mathrm{Satisfied}\;\mathrm{by}}{\leftarrow }\left[\begin{array}{ccccc} 1 & 0 & 0 & 0 & 0\\ 0 & 1 & 0 & 0 & 0\\ 0 & 0 & 1 & 0 & 0\\ 0 & 0 & 0 & 1 & 0\\ 0 & 0 & 0 & 0 & 1 \end{array}\right]\left\{\begin{array}{c} \mathrm{Olfactory}\;\mathrm{system}\\ \mathrm{Eyes}\;\mathrm{and}\;\mathrm{ocelli}\\ \mathrm{Petals}\\ \mathrm{Hair}\\ \mathrm{Floral}\;\mathrm{guides} \end{array}\right\}\\ \left\{\begin{array}{c} {\mathrm{FR}}_{31}\\ {\mathrm{FR}}_{32}\\ {\mathrm{FR}}_{33} \end{array}\right\}\overset{\mathrm{Satisfied}\;\mathrm{by}}{\leftarrow }\left[\begin{array}{ccc} 1 & 0 & 0\\ 0 & 1 & 0\\ 0 & 0 & 1 \end{array}\right]\left\{\begin{array}{c} \mathrm{Body}\;\mathrm{parts}\\ \mathrm{Pheromones}\\ \mathrm{Neural}\;\mathrm{components} \end{array}\right\}\\ \left\{\begin{array}{c} {\mathrm{FR}}_{331}\\ {\mathrm{FR}}_{332}\\ {\mathrm{FR}}_{333}\\ {\mathrm{FR}}_{334} \end{array}\right\}\overset{\mathrm{Satisfied}\;\mathrm{by}}{\leftarrow }\left[\begin{array}{cccc} 1 & 0 & 0 & 0\\ 0 & 1 & 0 & 0\\ 0 & 0 & 1 & 0\\ 0 & 0 & 0 & 1 \end{array}\right]\left\{\begin{array}{c} \mathrm{Round}\;\mathrm{dance}\\ \mathrm{Waggle}\;\mathrm{dance}\\ \mathrm{Duration}\;\mathrm{of}\;\mathrm{dance}\\ \mathrm{Sun}’s\;\mathrm{position}\;\mathrm{and}\;\mathrm{landmarks} \end{array}\right\} \end{array}$$

### 3.3. Information Axiom

In this study, we apply the information axiom to honeybees’ pollination process of apple flowers. The symbiotic relationship between honeybees and apple trees highlights the interdependence of species in maintaining healthy ecosystems and ensuring the global food supply. Apple trees belong to the Rosaceae family and are characterized by their beautiful and fragrant flowers. Each apple blossom consists of male and female reproductive parts. The male organs, called stamens, produce pollen, while the female organ, the pistil, contains the ovary that will develop into the fruit. For successful pollination, pollen grains must reach the pistil and fertilize the ovules. Honeybees are effective pollinators due to their unique characteristics and behaviors. Honeybees possess adaptations that make them highly efficient in transferring pollen from one flower to another. As honeybees forage for nectar, they unintentionally pick up pollen grains on their bodies, particularly on their hairy legs. When they visit another flower, some of the pollen rubs off onto the stigma, the receptive surface of the pistil, facilitating fertilization.

The relationship between honeybees and apple trees is mutually beneficial. Honeybees obtain nectar, their primary energy source, from the flowers. In gathering nectar, they unknowingly transfer pollen, allowing apple flowers to be fertilized. For apple trees, successful pollination is a prerequisite for fruit development. Without the transfer of pollen, apples would not form, resulting in diminished crop yields.

The information axiom complements the independence axiom by focusing on minimizing information content in designs, guiding how to choose among design alternatives that satisfy the independence axiom. The axiom is grounded in the belief that the best design is the one with the least amount of information, where “information” measures uncertainty or complexity in achieving the functional requirements of a design. The probability of a successful outcome quantitatively defines it. A lower information content indicates a more straightforward, more reliable design.

The information axiom is applied by calculating and comparing the information content of different design alternatives. This process typically involves three steps: (1) identifying the range of acceptable values (tolerance) for each FR; (2) determining the probability that a DP will fall within this acceptable range under normal operating conditions; and (3) calculating the information content based on this probability, using Shannon’s theory of information.

Given that *p_i_* is the probability that the design satisfies FR*_i_*, the information content *I_i_* of FR*_i_* is computed as follows [6]:

$$I_{i}=\log_{2}\frac{1}{p_{i}}$$

The design range is a designer’s tolerance spectrum, and the system range is the capability window. The intersection between the design range and system range is the common range. In the following, we illustrated how we can compute *p_i_* for the pollination process of apple flowers.

Honeybees are responsible for 90 percent of apple pollination. Hoffman et al. [38] examined where pollen is carried on the honeybee’s body and how it is transferred from one flower to the bee and finally to the stigma of another flower. A single contact between many pollen-carrying body parts and a stigma can transfer significant amounts of pollen. The study also revealed that bee behaviors unrelated to the intentional nectar and pollen collection, such as the frequent touching of stigmas by the claws, tarsi, or legs while foraging, grooming, and walking across flower clusters, could result in pollen transfer. These contacts occurred 65.9% of the time when a honeybee landed on a flower cluster. A mature standard apple tree with a heavy bloom may have as many as 100,000 flowers. Individual apple blossoms may be open for about 5 days, but the entire bloom period for the tree may range from 7 to 15 days. Only a tiny portion of these flowers, perhaps 8 to 10 percent, will eventually develop into fruits. One flower in a set of 20 will often be sufficient to produce a good yield. However, 15 to 20 percent is needed on trees that have sparse bloom. Each apple flower has about 20 anthers, and each anther produces about 3500 pollen grains (i.e., each flower produces about 70,000 pollen grains), although these numbers vary significantly in different varieties. A pollen grain is about 1/1000 inch in diameter (25.4 μm). An apple often has 10 seeds; however, varieties such as Jonagold may have only 3 or 4, and Northern Spy sometimes may have as many as 20. Jonathan, Honeycrisp, Gravenstein, Gala, Rome, Golden Delicious, Granny Smith, and Fuji apples usually have around five seeds. Theoretically, an apple flower of these species needs only 5 to 20 pollen grains from another variety to set a full complement of seeds. A fruit set is usually enhanced by providing massive quantities of foreign pollen. A bee carries about 100,000 pollen grains on its body and visits 10 or 15 flowers per minute or about 5000 flowers per day [39]. These data are used in the calculation of the common range.

**Figure 2:**
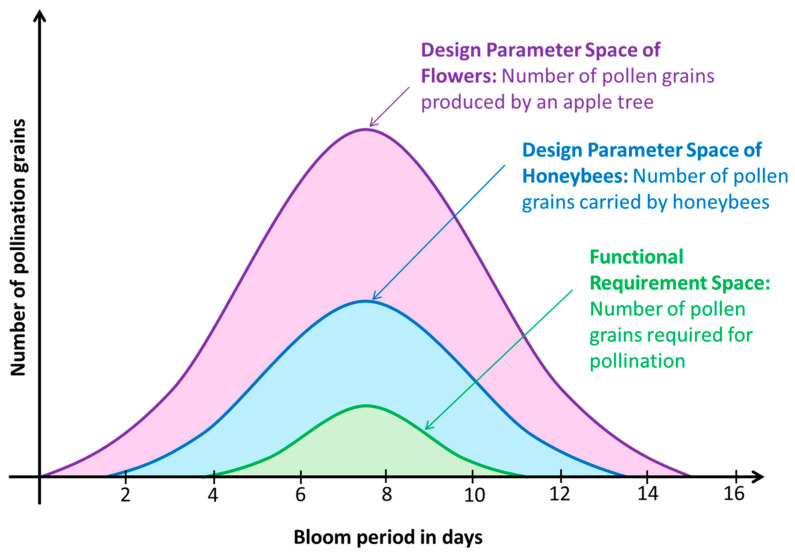
Functional requirement space, design parameter space of honeybees, and design parameter space of flowers, that enable the pollination process.

Figure 2 shows an approximate plot of the number of pollen grains available in a bloom period that may last about 15 days; this diagram is not drawn to scale. The *x*-axis shows the bloom period in days. The *y*-axis is the number of pollen grains available for pollination. Consider that the number of pollen grains produced in a bloom period follows a bell curve, and so do the number of pollen grains picked up and carried by honeybees and the number of pollen grains needed for pollination.

Let us apply the second axiom (i.e., the information axiom) of axiomatic design to the pollination process to check if it is robust. According to Way [39], a honeybee carries 100,000 pollen grains. Considering a factor of safety of 2, let us assume only half of them, i.e., 50,000 pollen grains on the bee’s body, are available for pollinating a flower. Let us also conservatively assume that the probability of a pollen grain on the bee’s body pollinating the flower is 0.001, i.e., 1 in 1000 opportunities. Consider each pollen on the bee’s body as a Bernoulli trial, and the success outcome is the successful pollination of the flower. From the literature [39], it is known that an apple flower of many species needs only 5 to 20 pollen grains from another variety to set a full complement of seeds. If *x* is the number of grains that successfully pollinate the flower, the probability that more than 20 grains successfully pollinate the flower is given by

$$P\left(x>20\right)=1-\sum_{x}^{20}b(x;\;\;50,000;\;\;0.001)=1$$

where *b*(*x*; 50,000; 0.001) represents the binomial distribution with 50,000 trials and a 0.001 probability of a successful outcome in a trial. Recall that FR_0_ = facilitate pollination process and DP_0_ = flower–honeybee mutualism. The information content of the pollination process designed to pollinate successfully is given by

$$I_{0}=\log_{2}\frac{1}{P(x>20)}=\log_{2}\frac{1}{1}=0$$

Since the information content of the pollination process design is close to 0, this design is simple, robust, and reliable; consequently, the pollination process has successfully produced seeds and fruits for millions of years. This longevity is enough to validate that the pollination process is robust.

## 4. Design Insights for Microrobots

A flower and a honeybee look like simple objects. However, they are complex and miniature structures with sophisticated anatomical and behavioral patterns that facilitate successful pollination processes. Honeybees, as a biological model, can offer a lot of clues for innovation. For example, bees use propolis, which is a sticky tree-resin substance, to seal their hive and protect the colony against pathogens. Despite its stickiness, honeybees can handle and manipulate propolis with their mandibles. An experiment conducted by Saccardi et al. [40] indicated that the medial surface of the mandible is covered with a fluid substance that reduces propolis adhesion. This design clue can be used to solve the problem of resin deposition on woodworking machine tools. The axiomatic design approach presented in this paper offers an elegant methodology for designing efficient microrobots. Microrobots and nanorobots are incredibly small, requiring specific design features to function effectively. Miniaturization allows them to navigate small spaces, interact with microscale objects, or access confined environments. They can be used for targeted drug delivery, minimally invasive surgeries, or exploring inaccessible areas within the human body.

Insect-inspired microrobots mimic the locomotion and behavior of insects, such as bees, wasps, or ants. They are often used for tasks like environmental monitoring, search and rescue operations, or exploration of narrow spaces. Micro aerial vehicles (MAVs) are tiny flying robots that can navigate and perform tasks in the air. They find applications in surveillance, aerial photography, and environmental monitoring. There are nanoscale robots that are constructed using DNA molecules as building blocks. They can perform specific tasks, such as molecular sensing or precise manipulation of biomolecules. Nano-swimmer robots are designed to move and navigate within liquids using various propulsion mechanisms. They can be used for tasks like cleaning contaminated fluids or nanoscale assembly. Kernbach et al. [41] described a meta-model of the re-embodiment of the biological aggregation behavior of honeybees in Jasmine microrobots in the context of the insect’s sensor–actor system.

The flower and honeybee are based on cradle-to-cradle designs and are made of biomaterials [42]. At the end of life, they decompose and become biological nutrients to other organisms in their ecosystem. These biomaterials are upcycled, i.e., decomposed into reused components to create a product of higher quality or value than the original [43]. The design of microrobots, which may be millions and billions in number in a few years, must embrace two design strategies: (1) cradle-to-cradle design and (2) upcyclable design. If these design strategies are not followed, the world may suffer from microrobot and nanorobot junk and pollution. These microrobots dispersed in oceans, land, skies, and space may lead to serious environmental damage. Disassembling and recycling big robots used in industrial processes is easier than end-of-life management of microrobots. If a product passport for every microrobot is made compulsory by legal statutes, and 100% recovery recycling plans are in place, future microrobot pollution can be avoided. Adisorn et al. [44] described a digital product passport (DPP) implementation strategy as a policy instrument for a circular economy.

The design features of flowers and honeybees and their interactions can offer many insights into the design of microrobots and nanorobots. Nature uses 4D printing technology to create organisms such as honeybees and flowers. Ahmed et al. [45] discussed the fundamentals, materials, applications, and challenging elements of 4D printing. Aldawood [46] reviewed various 4D printing applications in electronics, renewable energy, aerospace, food, healthcare, and fashion. For example, a small single-camera imaging system that provides a continuous 280-degree field of view (FOV) inspired by the large FOV of insect eyes. These innovations are well-suited for mobile robots, particularly for flying vehicles that need lightweight sensors [47]. This camera is made with metal, glass, acrylic, and composites. However, these technical materials are challenging to recover, reuse, and recycle. There were many innovations based on insects. For example, Shi et al. [48] presented a hydrodynamic model of honeybee mouth parts that may inspire the design of high-frequency microjoints for engineering applications, such as microstirrers.

Table 3 presents some insights we could use to design microrobots, taking inspiration from honeybees, flowers, and the pollination process.

Learnings from the pollination process in nature can inspire design principles for microrobots aimed at mimicking the efficiency and adaptability of natural pollinators. Some potential design principles are as follows: (1) Miniaturization: microrobots should be designed at a scale comparable to natural pollinators, allowing them to navigate through complex environments and access confined spaces within flowers. (2) Agile mobility: incorporate agile and versatile movement mechanisms to navigate through intricate floral structures, similar to the flight agility of bees and other pollinators. (3) Sensory systems: equip microrobots with advanced sensory systems, including cameras, proximity sensors, and environmental sensors, to detect and respond to changes in the surroundings, ensuring effective navigation and interaction with flowers. (4) Bio-inspired adhesives: develop adhesion mechanisms inspired by the attachment strategies of natural pollinators, allowing microrobots to adhere to different surfaces within flowers without causing harm. (5) Artificial intelligence: integrate artificial intelligence algorithms to enable autonomous decision making and learning from the microrobots, making them adapt to different floral structures and environmental conditions over time. (6) Material compatibility: ensure that the materials used in microrobot construction are biocompatible with easy and natural recycling and upcycling. (7) Environmental responsiveness: design microrobots to respond to environmental cues such as temperature, humidity, and light, similar to how natural pollinators adapt their behavior to changing conditions. By incorporating these design principles, researchers and engineers can develop microrobots that mimic the efficiency and adaptability observed in natural pollination processes, contributing to advancements in fields such as precision agriculture, environmental monitoring, and ecosystem preservation.

## 5. Discussion and Conclusions

Operations management is concerned with designing and controlling the production process and redesigning business operations to produce goods or services. Nature has also been operating using numerous and often efficient methods, such as pollination. The supply chain of a pollination process involves agents such as flowers and honeybees producing products such as nectar, pollen, honey, fruits, and seeds. The agents in nature’s processes often play dual roles as customers and suppliers.

The requirement for FRs to be satisfied independently by DPs underpins the goal of creating designs that are simple, efficient, and capable of meeting user needs without unnecessary complexity or interdependence. By adhering to the independence and information axioms, designers can create systems that are effective, adaptable, and robust against changes and uncertainties.

Some DPs perform multiple tasks. A kitchen knife is used for peeling, cutting, chopping, dicing, slicing, mincing, and separating tasks. Similarly, the mandibles of a honeybee on the head sides act like a pair of pliers. The mandibles are used for any chores in a hive, such as grasping or cutting wax to construct the comb, biting into flower parts (anthers) to release pollen, carrying detritus out of the hive, or gripping/biting enemies during nest defense. This looks like a coupled design. However, a careful analysis will reveal that the DP fulfills multiple FRs simultaneously. Honeybees use mandibles (a single DP) to satisfy different FRs at different times, not simultaneously. Therefore, it is not a coupled design but an ideal design.

Without pollination, the genetic diversity of plant species would decline, leading to an unstable ecosystem. This paper presented an elementary analysis of the pollination process by using axiomatic design technology. The FRs of the pollination process that have been discovered in the last hundred years or so (and many more are yet to be discovered), when mapped onto the DPs that nature has already created, the pollination design seems to be an “uncoupled design” which is the best design according to the theory of axiomatic design. However, the design equations presented in this paper are incomplete, and associations represent the high level of mapping of FRs and DPs. Much more research must be conducted to determine the specifications for all FRs and DPs. Only then can one conclude whether the pollination process is a good or suboptimal design. Because of the absence of specifications, the application of the second axiom (i.e., the information axiom) is rudimentary.

The axiomatic design methodology offers an elegant way of judging whether a design is good or suboptimal. In that sense, axiomatic design is a valuable tool. It can be applied to product design, service design, machine design, tooling design, software design, manufacturing system design, or logistics design. This paper focused only on the insights that can be derived from our observations and understanding of flowers, honeybees, and the pollination process to inspire microrobots and nanorobot designs. The field of microrobots and nanorobotics is still in its early research and development stage. However, they showcase potential applications and capabilities of miniaturized robots in various domains.

This study has its limitations. This study was possible only at a high level because nature’s pollination process is very complex with many interdependencies. This paper presents only an elementary analysis based on quantitative data presented by Way [39]. Within the limits of the data, this study is robust but incomplete. With advanced technologies such as the Internet of things (IoT) and video analytics researchers worldwide, the future might offer extensive data for studying the pollination process with axiomatic design.

## Figures and Tables

**Table 1:** The FRs and DPs of the communication system between a honeybee and a flower.

Level 0	Level 1	Level 2	Level 3	Design Parameters
FR_0_ = Facilitate pollination.				DP_0_ = Flower–honeybee mutualism.
	FR_1_ = Enable navigation. FR_2_ = Home in on specific feature of a flower. FR_3_ = Enable communication.			DP_1_ = Navigation system. DP_2_ = Localization system. DP_3_ = Communication system.
		FR_31_ = Mechanical communication. FR_32_ = Chemical communication. FR_33_ = Dance communication.		DP_31_ = Body parts. DP_32_ = Pheromones. DP_33_ = Neural components.
			FR_331_ = Location of food source near the hive. FR_332_ = Location of food source far from the hive. FR_333_ = Distance to the food source. FR_334_ = Direction of navigation.	DP_331_ = Round dance. DP_332_ = Waggle dance. DP_333_ = Duration of dance. DP_334_ = Sun’s position, Landmarks such as trees, buildings, etc.

**Table 2:** Master design matrix of the pollination process.

		DP_1_ Navigation System					DP2 Localization System					DP_3_ Communication System		
		DP_11_ (Eyes-1)	DP_12_ (Eyes-2)	DP_13_ (Internal Compass)	DP_14_ (Internal Clock)	DP_15_ (Odometer)	DP_21_ (Olfactory System)	DP_22_ (Eyes and Oocelli)	DP_23_ (Petals)	DP_24_ (Hair)	DP_25_ (Floral Guide)	DP_31_ (Body Parts)	DP_32_ (Pheromones)	DP_33_ (Neural System)
FR_1_	FR_11_	1	0	0	0	0								
	FR_12_	0	1	0	0	0								
	FR_13_	0	0	1	0	0								
	FR_14_	0	0	0	1	0								
	FR_15_	0	0	0	0	1								
FR_2_	FR_21_						1	0	0	0	0			
	FR_22_						0	1	0	0	0			
	FR_23_						0	0	1	0	0			
	FR_24_						0	0	0	1	0			
	FR_25_						0	0	0	0	1			
FR_3_	FR_31_											1	0	0
	FR_32_											0	1	0
	FR_33_											0	0	1

**Table 3:** Insights from the design features of flowers and honeybees in the pollination process.

Design Strategy	Flower	Honeybee	Insights
Cradle-to-cradle	Yes	Yes	Design a microrobot with a cradle-to-cradle strategy.
Material choices	100% biomaterials	100% biomaterials	Most microrobots use materials such as metal, glass, rubber, plastics, and composites. It would be good if biomaterials were selected at the design stage.
Power source (Power autonomy)	Rely on food produced by photosynthesis using sunlight.	Rely on food produced by plants that are 100% environmentally benign.	Usually, microrobots use chemical batteries that are difficult and costly to produce and recycle. Microrobots can be designed to operate on bio batteries charged by solar and wind energy.
Miniaturization	All flower parts are small, measuring a few micrometers to a few centimeters.	All parts of a honeybee are small, measuring a few micrometers to a few centimeters.	Miniaturization technology is emerging, and a lot of research has to be conducted.
Sensors	Biosensors	Biosensors	Biosensor technology is a well-researched topic, but a lot more to be advanced for their use in miniature engineered systems.
Actuators	Nature’s forces, like wind and sunlight, help flowers to move (e.g., sunflower)	Muscles made primarily of carbon.	Artificial muscles made of biomaterials need to be developed.
Manufacturing techniques	Nanotechnology, 4D printing.	Nanotechnology, 4D printing.	More advancement in 4D printing is required.
Upcycle	All flowers are upcycled and recycled 100% by nature.	All honeybees are upcycled and recycled 100% by nature.	The design strategy should include recycling, upcycling, and reverse logistics with a product passport for every microrobot for recovery, disassembly, segregation, reuse, and recycling.

## Data Availability

This article did not use any data except for a few statistics mentioned within the article.
